# Long-term effect of the Brazilian Workers’ Food Program on the nutritional status of manufacturing workers: A population-based prospective cohort study

**DOI:** 10.1371/journal.pone.0231216

**Published:** 2020-04-17

**Authors:** Karina G. Torres, Ingrid W. L. Bezerra, Gabriela S. Pereira, Raiane M. Costa, Anissa M. Souza, Antonio G. Oliveira

**Affiliations:** 1 Postgraduate Program in Health Sciences, Federal University of Rio Grande do Norte, Natal, RN, Brazil; 2 Nutrition Department, Health Science Center, Federal University of Rio Grande do Norte, Natal, RN, Brazil; 3 Pharmacy Department, Health Science Center, Federal University of Rio Grande do Norte, Natal, RN, Brazil; Universidad Nacional Autonoma de Mexico Instituto de Investigaciones en Ecosistemas y Sustentabilidad, MEXICO

## Abstract

**Background:**

The Brazilian Workers Food Program (WFP) is a public policy program of nutritional assistance to workers, with the main objective of improving nutritional conditions, which was implemented 40 years ago and serves over 21.4 million workers.

**Objectives:**

To compare the long-term change in anthropometric indicators of the nutritional status and dietary intake between workers of manufacturing industries adherent to and non-adherent to the WFP.

**Methods:**

A prospective cohort study, based on a combined stratified and multistage probability sampling, was carried out, with two waves with a 4-year interval. The change in body mass index (BMI), waist circumference (WC) and dietary intake at lunch by the 24-hour recall method were compared between groups with analysis of covariance.

**Results:**

A total of 273 workers in 16 industries from an initial cohort of 1069 workers in 26 industries of the State of Rio Grande do Norte in Brazil were evaluated in the two waves. The mean age was 37±10 years and 53.1% were male, with no differences between groups in age and sex distribution. BMI increased in both groups (0.44 kg/m2 in non-WFP, p = 0.003, and 0.56 kg/m2 in WFP, p = 0.0006) and WC increased in the WFP group (1.50 cm, p = 0.0006). BMI change over time did not show statistical differences between groups (p = 0.54) but WC had a greater increase in the WFP group (difference 1.37 cm, p = 0.047). There were no differences between groups in the change over time of the dietary intake.

**Conclusion:**

BMI and WC increased over time in manufacturing workers of industries both adherent and non-adherent to the WFP, but with a greater increase of WC in the WFP group. In order to achieve the objectives of the WFP, there will be a need for periodic evaluation and monitoring of nutritional indicators in these workers and implementation of monitoring and enforcement actions of the WFP.

## Introduction

The food consumption of workers has a fundamental influence on their health and quality of life, with repercussions on the overall perception of welfare and productivity at work. In the short term, the selection of healthy foods for nutritionally well-designed meals will promote improvements in stress reduction, accident prevention, attention levels, memory, learning, and productivity. In the long term, nutritional balance is directly related to the improvement of the nutritional status of individuals and populations, promoting advances in quality of life and reducing the prevalence of Chronic Noncommunicable Diseases (CNDs) [1].

In this sense, the development of food policies aimed at workers has become a relevant topic on the schedule of social initiatives in several countries. The implementation of policies for this public promotes benefits for governments, employers and employees in social, economic, tax and, particularly, in health aspects. Worldwide, there is large variation in scope and objectives across programs. While some countries encourage companies, public and/or private, to offer, in addition to meal breaks, an appropriate environment to conduct them, other countries encourage the use of meal vouchers supplied as paper or electronic cards. In this system, the employers usually pay between 50 and 100 percent of the meal vouchers and national laws determine the conditions and restrictions for using vouchers [2, 3].

In the early 1950s, the United Kingdom implemented the first worker’s food program. At first, the program was based on paper vouchers issued by each company and, in return, the British government granted tax exemptions. The model was so well accepted by companies and government that, from the 1960's on, several countries have also implemented the voucher system as a way of improving their workers' nutritional and health conditions. Currently, it is estimated that 46 countries encourage the use of meal vouchers, with over 47 million workers benefiting from this system, resulting in a positive impact on the economy and society [2, 4].

In Brazil, the right to health and food is a constitutional guarantee that is a fundamental part of the social rights of the population. Accordingly, public policies on food and nutrition have been developed and implemented through actions and programs designed to abolish food and nutrition insecurity among vulnerable groups of the Brazilian population, such as students and low-income workers. Several public policies aimed specifically at workers, representing strategic actions of health protection and promotion, have been implemented, among them the Occupational Health and Safety Policy, the Accident Risk Prevention Policy and the Workers' Food Program (WFP).

The WFP, established since 1976, is a food and nutritional assistance program for workers that, according to data from the Ministry of Labor, currently benefits 266.838 industries, covering 21.4 million workers. The main goals of the program, at the time of its creation, were to improve the nutritional conditions of workers and, secondarily, to improve the workers’ quality of life, to decrease the incidence of work accidents and to increase workers’ productivity [5].

Briefly, the WFP is a government initiative that uses tax incentives to encourage the employer to provide adequate food to their employees, prioritizing workers who receive up to 5 minimum wages, but extensible to workers with income above this limit. Any company can join the program, including individual microentrepreneurs, microenterprises, non-profit companies, as well as direct and indirect public administration bodies and entities. Once registered, the participation of the company in the WFP is automatically renewed, having continuous validity. The employee contributes an amount, limited to 20% of the costs, which may be the same for all employees or the company may choose to charge differently in proportion to the salary range. As a counterpart, adherent companies are exempt from labor, social security and tax charges on WPF expenses, thereby reducing employer expenses with their employees. In addition, the employer who opts for taxation based on the actual profit method may deduct WFP expenses up to 4% of the income tax due and in the event of an excess in the amount to be discounted, the difference may be deducted in two subsequent exercises. In practice, as smaller companies usually opt for the more tax-favorable presumed profit method, they are not eligible for the WFP.

The companies may be responsible for the whole process of producing and serving meals, may distribute food baskets, or may hire third party companies registered with the WFP to provide the service, which must offer a healthy and adequate diet to the energetic-nutritional demands of the assisted workers. Regardless of the method adopted for the provision of meals, the adherent company can offer its employees one or more meals a day, provided that the exemption limit is maintained. The nutritional parameters must be calculated based on the daily reference values for macro and micronutrients established in the WFP legislation.

However, the fact that workers have access to food at work in industries adherent to the WFP may not necessarily guarantee the consumption of a nutritionally balanced diet. Several studies in companies adherent to the WFP, on the one hand, have reported deviations of the diets provided from the regulatory directives with greater offer than recommended by the WFP on calories, protein, fiber, sodium, total fat and cholesterol, according to the nutritional assessments of the menus [6–8]. On the other hand, other studies have observed a higher prevalence of overweight and obesity among workers of WFP-adherent companies compared to non-adherent industries, suggesting a relationship to the food offered [9–11].

Considering the social and economic importance of this public policy focused on food security that has been implemented for over 40 years, studies evaluating the effect of the WFP on the nutritional and health status of workers become relevant. However, all such studies conducted so far have been cross-sectional surveys, which offer weak evidence of the role of the WFP in the nutritional status of workers. Therefore, we conducted a comparative study with the main hypothesis that, if the WFP is indeed associated with increased rates of overweight and obesity, then it is expected that workers in WFP-adherent companies have an increase over time in body mass index (BMI) and waist circumference (WC) that is higher than that observed among workers in non-WFP adherent companies. Thus, the aim of this study was to compare, between workers from manufacturing industries adherent and not adherent to the WFP, the change in indicators of nutritional status and food consumption at two evaluations with a 4-year interval.

## Methods

This was a prospective cohort study in workers from WFP-adherent and non-adherent manufacturing industries that were observed in two waves with an interval of 4 years in 2014 and 2018. The study was approved in both waves by the Institutional Review Board of the University Hospital Onofre Lopes, with the numbers 776.772/2014 and 2.087.237/2017 and conducted in accordance with the Declaration of Helsinki. All participants gave their informed consent in writing.

Two probability samples of workers, selected by combined proportional stratified and multistage sampling, were obtained from companies adherent and non-adherent to the WFP. Stratification factors were industry size with two levels: small (20 to 99 workers) and medium size (100 to 499 workers), and sector of industrial activity (alimentary, non-metallic minerals and textile). All small and medium-sized manufacturing companies in those sectors of activity located in the State of Rio Grande do Norte, Brazil, which consented to the research in writing were eligible for the study. Thirteen industries from each group (WFP and non-WFP) were selected by simple random sampling from all industries in the State at each combination of strata levels, in proportion to the total number of industries at that combination. In the second stage, 40 workers were selected by simple random sampling in each company included in the study. The inclusion criteria were: age over 18 years and at least one year working for the company. Pregnant women were excluded. The participants received no incentives to participate in the survey.

Background data collected in the first wave consisted of age, sex, marital status, number of children, highest level of formal education, wage and in-house specific training. The main study variables, two anthropometric indicators of nutritional status (Body Mass Index and waist circumference) were collected from the same workers in the two study waves. Before the study, the survey team was trained in the measurement of weight, height and waist circumference, according to the guidelines of the Brazilian Alimentary and Nutritional Surveillance System (SISVAN) [12]. BMI, defined by weight in kg divided by squared height in meters, was measured with a digital scale (Inner Scan, Tanita Corp., Tokyo) and a body height meter (Sanny, São Bernardo do Campo, SP, Brazil). WC, measured at the midpoint between the lower edge of the last rib and the iliac crest, was measured with a tape meter (Cescorf, Ltda, Porto Alegre, RS, Brazil). All measurements were made in duplicate and averaged.

Nutritional information on the food consumption of workers was obtained in both waves through a single 24-hour dietary recall (24HR), using the Automated Multiple Pass Method (AMPM), commonly used in population studies and which structures the collection of 24HR in stages [13]. The field data collections were conducted from Tuesday to Saturday to ensure that the description of the lunch consumed the previous day corresponded to that offered by the company, for workers from WTP companies. Information regarding food consumption was obtained by academic dietitians and supervised graduate students previously trained in alimentary interviews from each worker on all meals in the previous day, with specification of the time and the place where the meal was taken, in addition to the details of the preparation of the meals and the respective amounts expressed in home measures.

For nutritional analysis, the official 2011 Brazilian Table of Food Composition [14] was used, complemented when necessary by other food composition tables [15–17]. Values were calculated for total energy, macronutrients (protein, carbohydrate, total and saturated fat), sodium and fiber, which are the nutrients described in the regulations that define the nutritional parameters for WFP [18]. Nutrient consumption at lunch and at the remaining meals throughout the day was analyzed separately so that the consumption at lunch could be compared between workers from WFP-adherent and non-adherent companies.

In order to control for the level of workers physical activity, a validated Portuguese translation [19] of the short form of the International Physical Activity Questionnaire (IPAQ) [20] was used, as well as an assessment of the workers sedentary behaviour with the SIT-Q questionnaire [21]. The IPAQ is a self-administered questionnaire that asks about the amount of time spent in the previous 7 days in vigorous physical activity, moderate physical activity, walking and sitting, from which the total physical activity in Metabolic equivalent of task (MET) per minute per week. The SIT-Q questionnaire objectively quantifies the time spent daily with low energy expenditure activities in different domains, such as work, transportation, home and leisure.

### Statistical analysis

A sample size of 250 workers per group, WFP-adherent and non-adherent, would afford 90% power to detect a between-group difference greater than 0.3 kg/m^2^ (approximately 1 kg of body weight) in the change from the baseline in BMI, at the 5% significance level, assuming a standard deviation of the change from baseline in BMI of 1.2 kg/m^2^ [22]. It was estimated that about 50% of workers observed in the first wave would remain in the same industries in the second wave, so the sample size was set at 500 workers.

For comparison of the background characteristics of the workers between the two groups (WFP and non-WFP), chi-square and Student's t-tests were used. For comparison of the study variables (BMI, WC and nutrient intake) between the two waves within each group, paired t-test was used. For between-groups comparison of change from baseline in the study variables we used multiple regression adjusted by the initial value of the dependent variable (analysis of covariance), with the background variables that differed statistically between groups as covariates, adjusted by the scores of IPAQ and SIT-Q questionnaires, and stratified by sector of activity and company size. All tests were bilateral and a 5% level was adopted for statistical significance. Statistical analyses were performed with Stata 15 (Stata Corporation, College Station, TX, USA).

## Results

**Fig 1:**
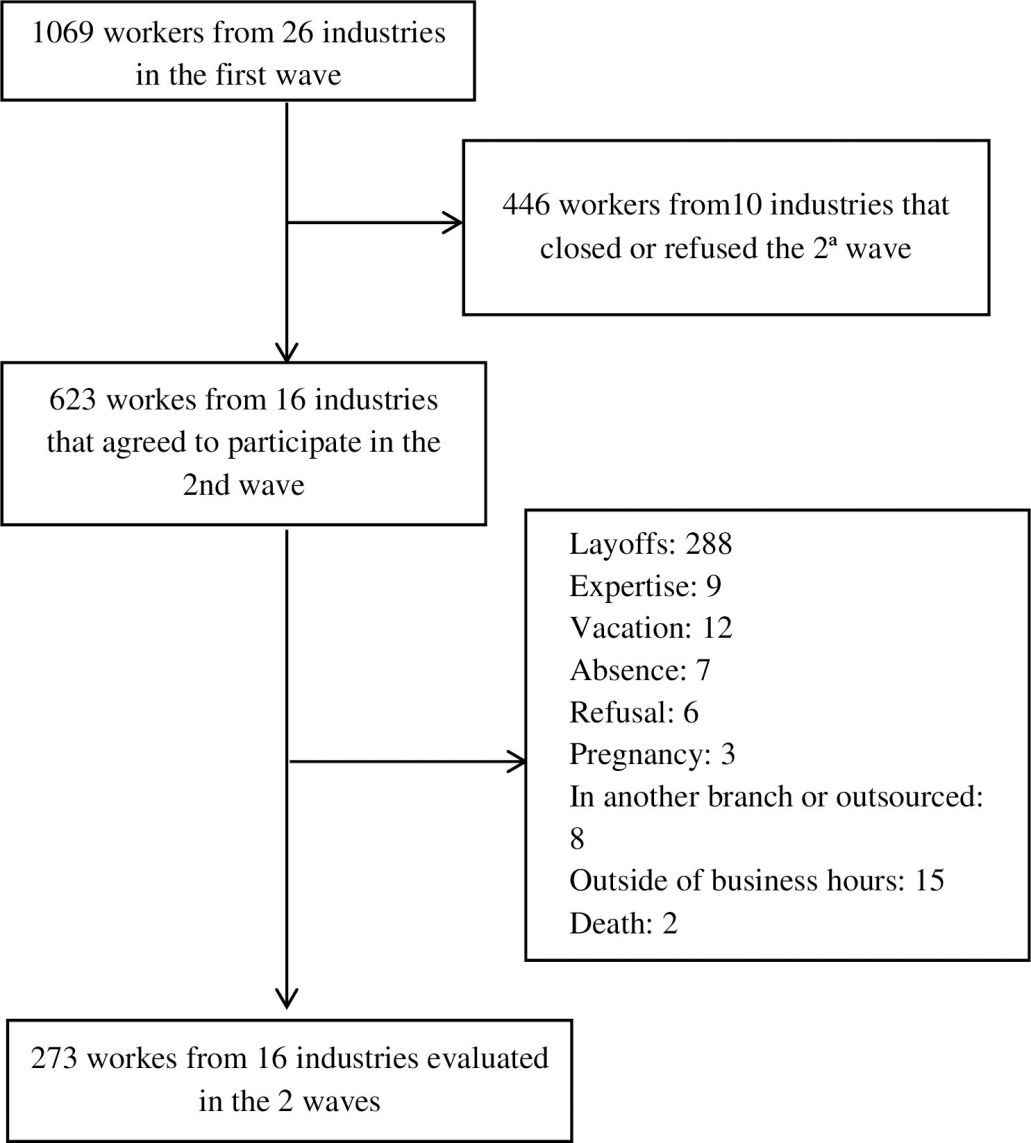
Disposition of the participants of the first and second waves of the study.

In the first wave of the study, conducted between September and December 2014, 1069 workers from a total of 26 industries were evaluated. Thus, in the second wave, 273 workers (25.5% of the initial cohort) were evaluated in 16 industries (61.5% of the initial sample). Among those, 143 workers were from WFP-adherent companies and 130 workers were from non-WFP-adherent companies. Fig 1 presents the disposition of individuals in the two waves of the study.

Of the 16 industries assessed in the second wave, eight were small and eight medium-sized, distributed across three sub-sectors of transformation industry: eight from the alimentary, six from the non-metallic minerals and two from the textile sector.

The average age of the workers was 37.2 ± 10.3 years, 53.1% were male and the average income was 1.43 ± 1.17 minimum wages. The groups were similar with respect to age and sex distribution, but workers in the WFP group had higher levels of education, income, job-specific training, and sedentary time (Table 1). There were no statistical differences in the initial characteristics (sex, income, professional qualification, BMI and WC) between the workers evaluated in the two waves and those evaluated only in the first wave, except for age that was slightly lower in the workers evaluated in the first wave only (34.5±11.1 years, p = 0.0004).

**Table 1:** Baseline characteristics of workers of manufacturing industry from the two cohorts adherent and non-adherent to the Workers’ Food Program in the State of Rio Grande do Norte, Brazil.

Variable	Non-WFP	WFP	Significance
	(n = 130)	(n = 143)	
Age	37.2 ± 11.3	37.2 ± 9.37	*t* = 0.03, df = 271, *p* = 0.98 *
Male sex	66 (50.8)	79 (55.2)	χ^2^ = 0.55, *p* = 0.46 **
Married	52 (40.0)	53 (37.1)	χ^2^ = 0.25, *p* = 0.62 **
Number of children	1.63 ± 1.67	1.50 ± 1.44	*t* = 0.71, df = 271, *p* = 0.48 *
Education ≥ high school	48 (36.9)	96 (67.1)	χ^2^ = 24.9, *p*<0.001 **
Income (minimum wages)	1.21 ± 0.49	1.62 ± 1.53	*t* = 2.97, df = 271; *p* = 0.003 *
Specific training	22 (16.9)	40 (28.0)	χ^2^ = 4.74, *p* = 0.030 **
Physical activity (METs/week)	1777 ± 1575	1950 ± 1624	*t* = 0.89, df = 271, *p* = 0.37 *
Sedentary time (min/day)	378 ± 186	426 ± 185	*t* = 2.12, df = 271, *p* = 0.035 *

Values are mean ± standard deviation or number (percentage)

*: Student’s t-test

**: chi-square test.

Table 2 shows the change over time of anthropometric indicators of nutritional status and of dietary intake at lunch and daily total. In the WFP group workers, there was a statistically significant increase in BMI (from 27.2 ± 4.48 kg/m^2^ to 27.8 ± 4.48 kg/m^2^, *t*-test, *t* = 3.50, df = 142, *p* = 0.0006) and WC (from 89. 4 ± 11.2 cm to 90.9 ± 11.5 cm, *t*-test, *t* = 3.50, df = 142, *p* = 0.0006). In the non-WFP group, only a statistically significant increase in BMI was observed (from 26.6 ± 4.61 kg/m^2^ to 27.0 ± 4.58 kg/m^2^, *t*-test, *t* = 3.01, df = 129, *p* = 0.003). There was no statistically significant change from baseline in total energy consumption and in nutrient intake in either group, except an increase in daily protein intake in the WFP group (from 368 ± 155 Kcal to 406 ± 160 Kcal, *t*-test, *t* = 2.43, df = 141, *p* = 0.016).

**Table 2:** Temporal variation of anthropometric indicators of nutritional status and dietary intake in workers from industries adherent and non-adherent from to the Workers' Food Program in the State of Rio Grande do Norte, Brazil.

	Non-WFP			WFP		
Year of observation	2014	2018	Sig. *	2014	2018	Sig.*
Variable	Mean ± SD	Mean ± SD		Mean ± SD	Mean ± SD	
**Nutritional status**						
BMI (Kg/m^2^)	26.6 ± 4.61	27.0 ± 4.58	*t* = 3.01, df = 129, *p* = 0.003	27.2 ± 4.48	27.8 ± 4.48	*t* = 3.50, df = 142, *p* = 0.0006
Waist circumference (cm)	88.1 ± 12.2	88.6 ± 12.2	*t* = 1.00, df = 129, *p* = 0.32	89.4 ± 11.2	90.9 ± 11.5	*t* = 3.50, df = 142, *p* = 0.0006
**Food consumption at lunch**						
Energy (Kcal)	742 ± 355	708 ± 329	*t* = 0.98, df = 126, *p* = 0.33	688 ± 319	683 ± 310	*t* = 0.15, df = 141, *p* = 0.88
Protein (Kcal)	183 ± 96	183 ± 87	*t* = 0.02, df = 126, *p* = 0.98	177 ± 85	190 ± 92	*t* = 1.35, df = 140, *p* = 0.18
Lipids (Kcal)	228 ± 181	212 ± 164	*t* = 0.84, df = 126, *p* = 0.40	198 ± 138	182 ± 121	*t* = 1.13, df = 140, *p* = 0.26
Carbohydrates (Kcal)	332 ±163	313 ± 160	*t* = 1.23, df = 126, *p* = 0.22	323 ± 169	312 ± 164	*t* = 0.68, df = 140, *p* = 0.50
Fiber (g)	14.4 ± 8.6	14.5 ± 8.3	*t* = 0.17, df = 126, *p* = 0.86	14.1 ± 8.5	14.8 ± 7.7	*t* = 0.91, df = 140, *p* = 0.36
Saturated fat (g)	7.9 ± 7.9	7.1 ± 7.0	*t* = 0.98, df = 126, *p* = 0.33	6.0 ± 5.0	5.4 ± 4.0	*t* = 1.24, df = 140, *p* = 0.22
Sodium (mg)	2030 ± 1215	2071 ± 1558	*t* = 0.29, df = 126, *p* = 0.77	1776 ± 889	1878 ± 1068	*t* = 0.97, df = 141, *p* = 0.33
**Daily food consumption**						
Energy (Kcal)	1978 ± 735	1903 ± 799	*t* = 0.99, df = 126, *p* = 0.32	1893 ± 746	1950 ± 626	*t* = 0.92, df = 141, *p* = 0.36
Protein (Kcal)	387 ± 194	374 ± 169	*t* = 0.68, df = 126, *p* = 0.50	368 ±155	406 ± 160	*t* = 2.43, df = 141, *p* = 0.016
Lipids (Kcal)	584 ± 303	547 ± 310	*t* = 1.10, df = 126, *p* = 0.27	534 ± 269	549 ± 240	*t* = 0.61, df = 141, *p* = 0.54
Carbohydrates (Kcal)	1009 ± 428	986 ± 410	*t* = 0.58, df = 126, *p* = 0.57	999 ± 436	995 ± 356	*t* = 0.10, df = 141, *p* = 0.92
Fiber (g)	23.8 ± 11.7	24.5 ± 11.6	*t* = 0.67, df = 126, *p* = 0.51	24.0 ± 13.9	24.1 ± 11.1	*t* = 0.11, df = 141, *p* = 0.91
Saturated fat (g)	22.4 ± 13.5	19.8 ± 13.2	*t* = 1.80, df = 126, *p* = 0.07	20.2 ± 12.0	19.3 ± 9.4	*t* = 0.85, df = 141, *p* = 0.38
Sodium (mg)	4262 ± 1864	4321 ± 2403	*t* = 0.27, df = 126, *p* = 0.79	3838 ± 1769	4187 ± 1886	*t* = 1.90, df = 141, *p* = 0.06

Sig.: significance; SD: standard deviation.

*: paired t-test.

The between-group comparison of the change over time in the anthropometric indicators of nutritional status and in dietary consumption is presented in Table 3. The increase in BMI was greater in the WFP group (0.14 kg/m^2^) but less than expected and the difference between groups did not reach statistical significance. The mean change from baseline in WC, however, showed a statistically significant difference between groups, increasing by an average 1.37 cm more in the WFP group than in the non-WFP group (multiple regression, *t* = , df = 263, *p* = 0.047). Regarding the change from baseline in nutrient intake, no statistically significant differences were observed between the groups.

**Table 3:** Change from baseline of the anthropometric indicators of nutritional status and dietary consumption in workers from industries adherent and non-adherent from to the Workers' Food Program in the State of Rio Grande do Norte, Brazil, and difference between groups.

Variable	Non-WFP	WFP	Difference	(95% CI)	Significance
	Mean ± SEM	Mean ± SEM			
**Nutritional status**					
Body Mass Index (Kg/m^2^) (%)	+0.44 ± 0.15	+0.56 ± 0.16	+0.14	(-0.32; +0.61)	*t* = 0.54, df = 263, *p* = 0.54
Waist circumference (cm)	+0.47 ± 0.47	+1.50 ± 0.43	+1.37	(+0.02; +2.71)	*t* = 2.00, df = 263, *p* = 0.047
**Food consumption at lunch**					
Energy (Kcal)	-34.3 ± 34.9	-4.8 ± 31.0	+9.40	(-68.6; +87.4)	*t* = 0.24, df = 258, *p* = 0.81
Protein (Kcal)	-0.20 ± 10.5	+12.8 ± 9.46	+6.94	(-15.6; +29.5)	*t* = 0.61, df = 258, *p* = 0.55
Lipids (Kcal)	-16.2 ± 19.2	-16.1 ± 14.3	-16.9	(-53.7; +19.9)	*t* = 0.90, df = 258, *p* = 0.37
Carbohydrates (Kcal)	-19.3 ± 15.7	-10.7 ± 15.7	-13.3	(-26.1; +52.7)	*t* = 0.66, df = 258, *p* = 0.51
Fiber (g)	+0.12 ± 0.71	+0.62 ± 0.68	+1.30	(-0.47; +3.07)	*t* = 1.44, df = 258, *p* = 0.15
Saturated fat (g)	-0.79 ± 0.81	-0.63 ± 0.51	-0.88	(-2.35; +0.59)	*t* = 1.18, df = 268, *p* = 0.24
Sodium (mg)	+41.8 ± 144.1	+101.4 ± 104.5	+2.31	(-321.8; +326.4)	*t* = 0.01, df = 258, *p* = 0.99
**Daily food consumption**					
Energy (Kcal)	-74.5 ± 74.9	+57.0 ± 61.9	+76.0	(-98.0; +250.0)	*t* = 0.86, df = 259, *p* = 0.39
Protein (Kcal)	-12.6 ± 18.5	+38.4 ± 15.8	+33.3	(-7.54; +74.2)	*t* = 1.61, df = 259, *p* = 0.11
Lipids (Kcal)	-37.3 ± 33.9	+14.9 ± 24.5	+4.63	(-66.0; +75.3)	*t* = 0.13, df = 259, *p* = 0.90
Carbohydrates (Kcal)	-23.8 ± 41.2	-3.62 ± 36.1	+22.0	(-71.8; +115.8)	*t* = 0.46, df = 259, *p* = 0.65
Fiber (g)	+0.68 ± 1.02	+1.33 ± 1.18	+1.08	(-1.59; +3.76)	*t* = 0.80, df = 259, *p* = 0.43
Saturated fat (g)	-2.63 ± 1.46	-0.89 ± 1.05	-0.58	(-3.52; +2.37)	*t* = 0.38, df = 259, *p* = 0.70
Sodium (mg)	+59.9 ± 221.9	+349.3 ± 184.4	+63.2	(-478.0; +604.4)	*t* = 0.23, df = 259, *p* = 0.81

SEM: Standard error of the mean. Multiple regression adjusted by the initial value of the dependent variable (analysis of covariance). P-values adjusted by baseline value, income (minimum wages), specific training, physical activity (Mets/week), sedentary time (minutes/day), company size (small/medium) and sector of activity (textile/non-metallic minerals/alimentary).

## Discussion

In the previously published analysis of first wave of this study [9], statistically significant differences have been shown between WFP and non-WFP groups, with higher rates of overweight/obesity and larger WC in workers of industries adherent to the WFP, after adjusting for differences in demographic characteristics among workers of the two groups. Thus, these results have shown an association between workers' access to food at work in companies adherent to the WFP and an increase in weight, an expected benefit of the program, but in a proportion of workers the increase was negatively affecting their nutritional status according to the measured anthropometric indicators.

The results now found in the second wave of the study showed that the anthropometric indicators of the nutritional status of the workers varied over 4 years, with a statistically significant increase in BMI in both groups and WC in the WFP group and that the increase in WC was greater in the WFP group, which supports the hypothesis of a causal nexus between worker participation in the WFP and increased WC and, possibly, BMI.

Other studies that evaluated the nutritional status of workers from companies adherent to the WFP also found high prevalence of overweight and obesity [23, 24]. In a cross-sectional study of 292 workers from industries adherent to the WFP located in the State of Paraná, southern Brazil, whose main objective was to verify whether the nutritional composition of the meals consumed at work correlated with energy intake and nutritional status of the workers, the prevalence of overweight and obesity was 47% in women and 61% in men, with 21.2% showing increased WC and 14.1% much increased WC. In another cross-sectional study of 4818 workers, randomly selected by multistage sampling from 157 companies across the country, the prevalence of overweight among WFP workers was 49.9%.

A classic study analyzing the impact of the WFP on workers nutritional status, developed by Veloso et al. [11] in the State of Bahia, based on retrospective data form about 8,000 workers, obtained from the database of a workers health monitoring service of that State, found that workers from companies adherent to the WFP had higher rates of overweight, higher serum triglycerides, total cholesterol and glycemia, and a higher prevalence of hypertension than workers from companies that did not offer any kind of food.

Regarding the analysis of the nutritional components of daily dietary intake, a statistically significant increase was observed between the two waves in workers of the WFP group in protein intake and, possibly, also in sodium intake. The other components of food consumption did not present statistical differences over time. The purpose of the analysis of the nutritional components of dietary intake was to find an explanation for an eventual weight increase among workers from WFP-adherent companies. However, statistically significant differences between groups in nutrient intake at lunch and daily total could not be found, presumably because of the large variation of nutrient intake resulted in a study that was not powered to demonstrate such differences. Only one previous study evaluating the WFP has presented a comparative analysis of nutrient intake, but limited to consumption during lunch, having shown a lesser intake of saturated fat and sodium among workers from WFP-adherent companies [9].

The main contribution of the present study was to produce further evidence of a causal link between the WFP and overweight in manufacturing workers by showing a temporal relationship between WC increase and WFP adherence. This fact in itself is worrisome and compromises some of the WFP's main objectives of promoting workers' health and welfare.

It is important to emphasize that adequate nutrition is a basic requirement for health promotion and protection, being recognized as a determinant and necessary factor for the health of individuals and communities. Therefore, the WFP is a relevant example of a public policy whose role is to guarantee food and nutrition security for workers, based on the principle of food sovereignty and the right to quality and sufficient quantity of food without compromising other essential needs. Actually, over the 40 years of its existence numerous studies evaluating the WFP have found a significant decrease in the prevalence of malnutrition among Brazilian workers, which proves its relevance, both from a nutritional and from a social perspective. It must not be overlooked that it is also the role of public food policies the promotion of healthy eating practices that respect cultural diversity and that are environmentally, economically and socially sustainable. However, the results of this study have revealed that, in its present state, the WFP may be deviating from those objectives and having undesirable effects on the nutritional profile of workers. These, however, may be reversed through a better planning and close monitoring of the food offered by the program.

Obesity has a multifactorial cause and its increasing prevalence is being attributed to several biopsychosocial processes. The “environment” (political, economic, social and cultural aspects), and not only the individual and his choices, assumes a strategic place in the analysis of the problem and in the interventions proposed [25]. Therefore, the development of food and nutrition education actions near workers in the settings where meals are offered becomes essential. In this sense, national laws, in line with the International Covenant on Economic, Social and Cultural Rights, recommended the development of such activities directed to the clientele served by the WFP. In fact, favorable results have been found through the adoption of diversified strategies and interventions involving dietary orientations and individual and group physical activities in the workplace [26].

In addition, the WHO believes that the workplace should provide the opportunity and encourage workers to make healthy choices. Being a public policy whose primary role is the provision of food based on the principles of adequacy and health, according to Global Strategy on Diet, Physical Activity and Health [27], its implementation aims to ensure access to a balanced and planned diet according to the nutritional needs of the workers. We believe that educational actions in the workplace have an important role in the promotion of changes in daily food consumption and eating habits and must be considered as an important complement of the WFP in its role for the strengthening of the food and nutrition security of this population.

This study has some limitations. The survey was conducted in only three subsectors of the manufacturing industry and in small and medium-sized companies, but the selected sectors and company sizes are the most productive in the manufacturing industry. The study was conducted in a single State and its results may not reproduce in other regions. However, there has been no evidence that the relationship between WFP and nutritional status is different in other States, considering that studies conducted in different locations have shown similar results. The 24-hour recall has limitations due to the possible memory bias of food consumption due to the collection of retrospective data, but it is well suited for epidemiological research that needs to recruit a large number of individuals in a short period of time. The study had been sized with adequate power to detect a slight difference in BMI between the groups, but it was only possible to obtain data from half of the initially expected number of workers. This was due to the closure of a considerable number of companies as a result of the economic crisis that began immediately after the completion of the first wave and lasted throughout the study period. However, there were no significant differences in demographic data in workers not evaluated in the second wave, except for a slight difference in mean age.

On the other hand, the probability sampling, the longitudinal design of the study and the large sample size are aspects that strengthen the results. This is the first study with direct observation, in prospective and comparative cohorts between workers from companies adherent and non-adherent to the WFP, representing in our view a significant contribution to the evaluation of this program.

For future research, it would be important to conduct further longitudinal studies in other States to confirm the temporal relationship between workers participation in the WFP and the change over time in anthropometric indicators of the nutritional status. In addition, the qualitative and quantitative aspects of the menus implemented in companies adherent to the WFP could be analyzed in order to evaluate the level of adequacy of the food offered to workers with the nutritional parameters defined by the WFP.

The findings of this study strengthen recommendations for the implementation of periodic monitoring of nutritional indicators in this population of workers, as well as educational actions for healthy nutrition ministered in the workplace that encourage the adoption of healthier and more active lifestyles as a health promotion strategy and in order to improve the workers’ quality of life. As the WFP is a public policy focused on food and nutrition security, making adjustments in the offered meals can be an important measure to promote workers health.

## Conclusion

In evaluating the long-term evolution of anthropometric measures of nutritional status and of food consumption, it was found that workers from industries adherent to the WFP showed a more pronounced increase in WC. This finding supports the hypothesis that the higher BMI and WC, and higher prevalence of overweight and obesity, that have been described in the literature in workers from WFP-adherent companies are actually due to their permanence in this program. This demands for the need for a better supervision and monitoring of the execution of the program in order to adjust the nutritional quality of the meals offered and, consequently, to obtain better results regarding the nutritional status of the workers.

## Supporting information

S1 Dataset (XLSX)
